# Annual Research Review: Interventions for young children exposed to trauma

**DOI:** 10.1111/jcpp.70121

**Published:** 2026-01-19

**Authors:** Katherine L. Guyon‐Harris, Kathryn L. Humphreys

**Affiliations:** ^1^ University of Pittsburgh School of Medicine Pittsburgh PA USA; ^2^ Vanderbilt University Nashville TN USA

**Keywords:** Trauma, intervention, infant mental health, early life experience, caregiver

## Abstract

The landscape of trauma‐focused interventions for young children has evolved significantly, though substantial gaps remain. Early childhood trauma exposure occurs during sensitive periods of brain development with potential lifelong consequences. However, these periods also present unique opportunities for intervention to redirect trajectories toward positive outcomes. Rapid neurodevelopmental changes across early childhood necessitate interventions specifically designed for evolving capacities rather than simply “scaled down” versions of adult treatments. A review focused exclusively on evidence‐based interventions for young children is needed. This review represents a synthesis of the literature informed by our clinical and research expertise. We review interventions that (1) target trauma symptoms as primary outcomes, (2) were designed for children ages 0–8 years, (3) include substantive caregiver involvement, and (4) have empirical support from published randomized controlled trials or well‐designed quasi‐experimental studies. Our review revealed a tiered evidence base for young children, with the strongest support for interventions targeting specific age groups: Child–Parent Psychotherapy for infants and toddlers, Preschool PTSD Treatment for preschoolers, and Trauma‐Focused CBT for early elementary children. Critical gaps include limited interventions for children under age 3, sparse evidence for interventions targeting noninterpersonal trauma, assessment challenges, particularly with longitudinal measurement across developmental transitions, and insufficient implementation research on disseminating interventions in community settings. By continuing to refine effective trauma interventions for our youngest children, we can alleviate immediate suffering and potentially prevent decades of associated difficulties across the lifespan. Future research priorities should include expanding the evidence base for existing interventions through well‐powered trials with diverse samples, developing and testing preventive interventions delivered following potentially traumatic events, adapting established interventions for under‐studied trauma types, and implementation research to support widespread adoption in real‐world settings.

## Introduction

The experience of traumatic events at any point across the lifespan can be debilitating. Children experience reactions following trauma exposure and require intervention similar to adults, yet this understanding has only gained traction over the past few decades (Chu et al., 2025; Meiser‐Stedman, 2002). Recent comprehensive reviews have similarly emphasized the importance of age‐appropriate, developmentally informed approaches across early childhood (Willheim & Schechter, 2025). While research on trauma among older children and adolescents has expanded significantly, empirical and theoretical work on trauma in young children—particularly infants, toddlers, preschoolers, and early elementary children (under age 8 years)—lags considerably behind.

The dramatic neurocognitive development during early childhood (Salmon & Bryant, 2002) makes understanding trauma experiences in young children more complex than in older populations with more developed abilities. For young children, trauma experiences can have particularly profound impacts due to their rapidly developing brains and heightened vulnerability (Perry, Pollard, Blakley, Baker, & Vigilante, 1995). In the aftermath of trauma, they remain entirely dependent on adult caregivers for safety, protection, and access to intervention. Consequently, interventions for young children necessarily include caregivers in the therapeutic process.

This review addresses a significant gap in the literature by examining interventions specifically designed for trauma‐exposed children ages 0–8 years. This review represents a synthesis of the literature informed by our clinical and research expertise rather than a systematic or scoping review following formal search strategies such as PRISMA guidelines. While several reviews have examined trauma interventions broadly across childhood and adolescence, there is a need for a review focused exclusively on evidence‐based treatments for the earliest developmental period, with particular attention to caregiver involvement requirements. We begin by defining trauma in early childhood, followed by theoretical frameworks guiding intervention approaches. We then examine evidence‐based interventions organized by developmental periods, highlighting exemplar treatments with demonstrated efficacy. Finally, we discuss innovations, challenges, and future directions, with specific attention to assessment issues, emerging treatment approaches, and gaps in research for noninterpersonal trauma.

## Definitions of trauma and developmental impacts

Traumatic events include experiences that are interpersonal (e.g., physical abuse, sexual abuse) or noninterpersonal (e.g., motor vehicle accident, natural disasters) that are experienced as life‐threatening. Experiences of trauma threaten fundamental human expectations of safety and security. Traumatic experiences that are interpersonal in nature can additionally impact fundamental human needs for belonging and trust. Both trauma types can cause an array of symptoms, including posttraumatic stress disorder (PTSD).

In this review, we focus on “criterion A” events—serious, life‐threatening events likely to cause trauma‐related symptoms. We exclude events that may be distressing but not life‐threatening (e.g., divorce, loss of a loved one) and experiences of psychosocial neglect, which represent fundamentally different experiences requiring different intervention approaches. We also exclude complex trauma,^1^ which represents a distinct theoretical and clinical framework (Cook et al., 2005) that falls outside this review's scope.

The understanding that young children can experience trauma and develop PTSD similar to adults has evolved gradually. While there has been general acceptance since approximately 1980 that children ages 6 years and older experience trauma and benefit from treatment, recognition for children ages 5 years and younger has lagged significantly. Specialized developmental criteria for PTSD among young children were not included in the Diagnostic and Statistical Manual of Mental Disorders until its fifth edition in 2013 (American Psychiatric Association, 2013; McKinnon et al., 2019).

Early childhood represents a foundational period where multiple developmental systems experience sensitive periods for optimal growth. Trauma exposure during this time can alter lifelong development across multiple domains (Andersen & Teicher, 2008). Internalizing problems in adulthood vary by age of onset of childhood maltreatment, with greater prevalence when maltreatment occurs between ages 0 and 5 years (Kaplow & Widom, 2007).

Thus, young children's dependency on caregivers creates unique vulnerabilities to trauma. All validated interventions for trauma‐exposed young children require caregiver involvement to varying degrees—a requirement not as essential for interventions with older populations. Young children rely on caregivers not only for physical protection but also for developing foundational self‐regulation skills. For these reasons, interventions for children ages 0–8 years are distinctively important and constitute the focus of our review.

## Prevalence of trauma exposure

Young children, particularly infants under age 1 year, have disproportionately high rates of trauma exposure compared to older children and greater vulnerability to serious injuries or fatalities. In the United States, nearly 4.4 million referrals involving over 7.7 million children were made to child protective services in 2023 (U.S. Department of Health and Human Services, 2025). Infants (birth to age 12 months) experience the highest victimization rate at 21.0 per 1,000 children. Alarmingly, 44% of child fatalities from abuse and neglect occur among infants under 1 year (U.S. Department of Health and Human Services, 2025).

Global statistics follow similar patterns. WHO estimates suggest nearly 300 million children aged 2–4 years worldwide regularly experience violent discipline from caregivers, and 1 in 4 children under age 5 live with a mother who experiences intimate partner violence (WHO, 2020). These statistics highlight that early childhood trauma represents a global public health crisis.

Data on noninterpersonal trauma exposure (e.g., motor vehicle accidents, natural disasters, medical trauma) is less comprehensive, particularly when disaggregated by age. This data gap significantly limits understanding of the full scope of trauma exposure among young children and highlights the need for more systematic documentation of noninterpersonal traumatic experiences across this critical developmental period.

## Theoretical models of traumatic exposure

Several theoretical frameworks conceptualize the impact of trauma on young children. We review four prominent perspectives that provide complementary understandings of trauma's effects and guide intervention strategies.

### Learning and behavioral theory

From a learning perspective, trauma in young children can be understood through fear conditioning, whereby traumatic events serve as unconditioned stimuli, and previously neutral stimuli become associated with threat. Environmental stimuli that were once benign become trauma reminders (including places, objects, individuals, and sensory input). Given their developing cognitive abilities and fundamental dependence on primary attachment figures for emotional and psychobiological regulation, young children are particularly vulnerable to fear conditioning and often display overgeneralized fear responses (Scheeringa & Zeanah, 2001).

#### Intervention implications

This framework directly informs exposure‐based intervention components, where children are gradually exposed to trauma reminders in safe contexts to reduce fear responses. Techniques such as systematic desensitization and in vivo exposure are postulated to help children develop new, nonthreatening associations with previously feared stimuli (Cohen, Mannarino, & Deblinger, 2017).

### Cognitive models

A prominent cognitive model is the just world theory hypothesis (Janoff‐Bulman, 1992). Children are born with fundamental assumptions that the world is safe and fair. Traumatic experiences can lead individuals to question these beliefs. To manage the terrifying concept that the world is not safe, young children often blame themselves or others for traumatic events because, if someone is at fault, the belief that the world is just can be restored. Consequently, maladaptive beliefs about the self, others, and the world are common in trauma‐exposed young children (Meiser‐Stedman, 2002).

#### Intervention implications

Cognitive processing components address these maladaptive beliefs by helping children develop more adaptive thought patterns through gentle challenging of self‐blame, age‐appropriate psychoeducation about trauma impacts, and guided meaning‐making activities (De Arellano et al., 2014). For individuals of all ages, particularly among young children, these techniques help counter cognitive distortions that commonly emerge following trauma exposure.

### Developmental theory

Developmental theory suggests that trauma in early childhood disrupts normal development processes, particularly during sensitive periods. Trauma's impact varies based on the child's developmental stage at exposure. Given the concentration of sensitive periods during infancy and toddlerhood, trauma during this period can have severe cascading effects on brain architecture, stress response systems, and the development of essential executive functions (Gunnar & Quevedo, 2007; Perry et al., 1995; Schore, 2001).

#### Intervention implications

Developmental considerations inform age‐appropriate intervention strategies, with play‐based approaches emphasized for younger children and more verbal processing introduced with older children. Interventions often include developmental guidance for caregivers and focus on rebuilding competencies disrupted by trauma.

### Attachment theory

Attachment theory underscores caregivers' importance in buffering children from trauma effects and explains the particularly destructive impact of caregiver‐perpetrated trauma. The presence of at least one secure attachment figure can protect a child from trauma's negative impacts (Feldman & Vengrober, 2011). Conversely, when caregivers perpetrate trauma, development is severely affected (Cicchetti & Toth, 2016). Trauma within the caregiver–child relationship often leads to disorganized attachment (Main & Solomon, 1990), where children experience “fear without solution” as the very person they seek for protection becomes a source of threat.

#### Intervention implications

Attachment theory forms the foundation for dyadic interventions focused on the caregiver–child relationship, with goals to rebuild trust, promote caregiver responsiveness, and foster secure attachments.

### Integration of theoretical models in trauma intervention

These theoretical frameworks—learning/behavioral, cognitive, developmental, and attachment—provide complementary perspectives on how trauma affects young children and inform different aspects of intervention. While no single framework can fully capture the complex impact of trauma on developing children, together they create a comprehensive foundation for understanding and addressing trauma in this uniquely vulnerable population.

The integration of these frameworks is evident in how effective interventions are structured across different developmental stages. For infants and toddlers, attachment theory predominates, with interventions focusing heavily on the caregiver–child relationship. Learning theory principles are applied primarily through the caregiver's responses to the child, helping to create new, nonthreatening associations with trauma reminders. Cognitive elements are addressed indirectly through the caregiver's provision of safety, predictability, and affirmation.

For preschoolers, while attachment remains crucial, learning theory begins to take a more direct role as children engage in graduated exposure activities. Cognitive elements emerge as children develop capacities to express and process simple thoughts about their experiences, though still heavily scaffolded by adults. Developmental theory guides the use of play and drawing as primary therapeutic modalities.

For early elementary children, cognitive models gain prominence as children develop increased capacity for verbal processing and understanding cause–effect relationships. Learning theory continues to inform exposure components, with children taking a more active role in identifying triggers and practicing coping skills. Attachment theory remains important but shifts toward supporting the child's growing autonomy while maintaining the secure base provided by caregivers.

This developmental progression from predominantly attachment‐based to increasingly cognitive‐behavioral approaches reflects how the expression and processing of trauma shifts across early childhood. The most effective interventions integrate elements from all four frameworks, calibrated to match the child's developmental capacities and needs. This integration acknowledges that trauma affects multiple domains simultaneously—neurobiological, cognitive, emotional, behavioral, and relational—necessitating a comprehensive approach to healing.

## Evidence‐based interventions for young children exposed to trauma

Several core therapeutic elements (see Table 1) emerge from the theoretical frameworks as essential components of effective trauma interventions for young children. While implemented differently across age groups and intervention models, these components form the foundation of evidence‐based trauma treatment and address specific aspects of trauma impact. In addition to these core elements, interventions are supported by *education and preparation*, also termed *psychoeducation, in which families are* provided information about trauma, its effects, prevalence, and expected treatment outcomes. This component builds engagement and rapport, normalizes trauma experiences, validates emotions, and reduces stigma. For young children, psychoeducation is delivered primarily through caregivers, with simple, concrete explanations provided directly to children based on their developmental capacity.

**Table 1:** Core elements of trauma‐focused interventions for young children

Core intervention element	Theoretical foundation	Infants/Toddlers (0–2)	Preschoolers (3–5)	Early elementary (6–8)
Safety and stabilization	Attachment, Learning	Establishing predictable routines; Promoting caregiver co‐regulation	Creation of safety plans; Teaching simple calming techniques	Collaborative safety planning; Teaching more complex self‐regulation skills
Exposure	Learning, Behavioral	Therapist‐guided play with trauma‐relevant toys	Storytelling and drawing about trauma experiences	Structured trauma narrative creation; More direct verbal processing
Cognitive processing	Cognitive	Caregiver provides verbal affirmations of safety and worth	Basic exploration of child's understanding of what happened	More explicit work addressing thoughts about self, others, and trauma
Skill building	Developmental	Focus on co‐regulation and attachment security	Teaching basic emotional identification and simple coping strategies	More sophisticated emotion regulation and problem‐solving skills
Caregiver involvement	Attachment	Caregiver as primary therapeutic agent; Always present in sessions	Combined individual child‐only and joint sessions; Caregiver as observer and participant	Caregiver as supporter and reinforcer; More independent child work with joint processing

### Intervention elements

Interventions for trauma‐exposed young children address PTSD symptoms including re‐experiencing, arousal, negative affect states, and numbing or dissociation. While many components parallel those in treatments for older populations, the degree of caregiver involvement uniquely distinguishes interventions for young children.

*Safety and stabilization* form the foundation of trauma treatment. Before processing trauma content, physical and emotional safety must be established (Cohen et al., 2017). Many interventions specifically contraindicate treatment during ongoing trauma (e.g., continued abuse, active domestic violence). Once safety is secured, establishing predictable routines and building emotional regulation skills create the foundation for further trauma work.

*Exposure* is fundamental to trauma‐focused therapies. For young children, exposure occurs through developmentally appropriate activities like play, storytelling, or art. These modalities allow children to process trauma narratives in manageable doses, with therapists selecting materials that facilitate trauma exploration (e.g., medical equipment for children processing medical trauma).

*Cognitive processing* addresses maladaptive beliefs about self, others, and the world that commonly develop following trauma. For verbal children, this includes age‐appropriate education about trauma's effects and gentle correction of misattributions. With preverbal children, this may involve caregivers providing affirming messages about the child's worth and safety. It is important to note that for very young children, cognitive approaches must be scaffolded through caregivers and play‐based activities. The applicability of cognitive models decreases with younger ages, and for infants and toddlers, attachment‐based and relationship‐focused approaches may be more developmentally appropriate.

*Skill building* supports the development or restoration of age‐appropriate competencies that trauma may have disrupted. Key skills include emotional identification and regulation, coping strategies, social skills, and problem‐solving.

*Caregiver involvement* varies systematically across development while remaining essential throughout early childhood. The nature and extent of caregiver involvement must be carefully considered based on the caregiver's role in the trauma and current safety factors. When caregivers have perpetrated trauma, specialized clinical judgment is required to determine appropriate involvement levels, with child safety remaining paramount.

- For infants and toddlers (0–2 years), caregivers are primary therapeutic agents, present in all sessions, with interventions enhancing caregivers' ability to provide sensitive care and co‐regulation.
- For preschoolers (3–5 years), involvement balances between observation, joint sessions, and supporting the child's emerging therapeutic relationship with the clinician.
- For early elementary children (6–8 years), caregivers focus on reinforcing skills, participating in joint processing sessions, and maintaining consistency across environments.

## Evidence‐based interventions by developmental period

Despite clear evidence that young children experience trauma at high rates and develop PTSD, evidence‐based interventions specifically designed for children under 8 years remain limited compared to treatments available for older populations. We review interventions (see Table 2) that (1) target trauma symptoms as primary outcomes, (2) were designed for children ages 0–8 years, (3) include substantive caregiver involvement, and (4) have empirical support from published randomized controlled trials or well‐designed quasi‐experimental studies.

**Table 2:** Interventions for young children exposed to trauma

Intervention	Age range	Primary trauma types	Treatment structure	Core components	Empirical support
Child–Parent Psychotherapy	0–6 years	Domestic violence, child maltreatment, other interpersonal trauma	Dyadic sessions with caregiver and child Weekly 60‐min sessions Duration: 6–12 months (avg. 32 sessions)	Play‐based Attachment‐focused Trauma processing through relationship Developmental guidance Emotion regulation	5+ RCTs Largest sample: *n* = 199 Follow‐up: 6–12 months Outcomes: PTSD symptoms, attachment, behavior problems, physiological regulation
Preschool PTSD Treatment	3–6 years	Various trauma types including natural disasters, domestic violence, accidents	Individual sessions with child (caregiver observes) Joint sessions (1, 2, 12) 12 weekly 45‐min sessions	Psychoeducation Emotional identification Coping skills Graduated exposure (drawing, imaginal, in vivo) Safety planning	1 RCT (*n* = 64) Follow‐up: 6 months Effective across trauma types Outcomes: PTSD symptoms, depression, separation anxiety
Trauma‐Focused CBT (TF‐CBT)	3–18 years (evidence for 3–8)	Sexual abuse, physical abuse, domestic violence, various trauma types	Parallel child and caregiver sessions Joint sessions in later phase 12–16 weekly sessions	Psychoeducation Coping skills Emotional processing Trauma narrative Cognitive processing Behavior management	10+ RCTs with young children Samples up to *n* = 210 Follow‐up: up to 12 months Outcomes: PTSD symptoms, depression, behavior problems, caregiver distress
Stepped Care TF‐CBT	3–7 years	Various trauma types including accidents, domestic violence, community violence	Step 1: Caregiver‐led home sessions with minimal therapist support Step 2 (if needed): 9 standard TF‐CBT sessions	Same as TF‐CBT, with Step 1 delivered primarily by caregivers Skill mastery determines progression to Step 2	1 RCT (*n* = 53) Follow‐up: 3 months Noninferior to standard TF‐CBT More effective for internalizing than externalizing symptoms

We have excluded interventions that were designed for broad age ranges (e.g., 6–18 years) where the average participant age consistently fell outside our 0–8 years focus, indicating that young children were not the primary target population. Also excluded are trauma‐informed approaches that address general behavior problems or psychopathology rather than targeting trauma symptoms directly, as well as general psychotherapy approaches without specific trauma components, parenting interventions without trauma focus, and school‐based universal prevention programs.

### Interventions for infants and toddlers

Of all the age groups reviewed, exposure to trauma among infants and toddlers and subsequent interventions may be one of the least understood. A persistent challenge is the long‐held assumption that infants and toddlers are less impacted by trauma due to immature memory and cognitive processing systems. Although infants and toddlers may not share the same capacity for verbal processing of trauma, decades of evidence clearly dictate that they are profoundly impacted by trauma exposure (Scheeringa, Zeanah, Myers, & Putnam, 2003).

Infants and toddlers are especially reliant on caregivers for basic survival as well as stabilization and emotional regulation following trauma. Interventions for this age group are necessarily dyadic, with a focus on helping the child through the caregiver–child relationship.

*Child–Parent Psychotherapy (CPP)* is the most well‐established intervention for treating trauma in infants and toddlers. This dyadic, attachment‐based intervention targets the relationship between child and caregiver in order to acknowledge, prevent, and recover from trauma within the dyad (Lieberman, Ghosh Ippen, & Van Horn, 2015). Originally designed to treat exposure to domestic violence, it has been successfully applied to various interpersonal traumas.

Sessions include the dyad or caregiver alone (never the child alone), focusing on developmental guidance to encourage developmentally appropriate interactions between caregiver and child. Sessions also focus on building emotional and behavioral regulation with attention to both coregulation and emerging independent regulation.

The intervention proceeds through three phases: (1) foundational assessment and engagement, where the clinician conducts an assessment to guide treatment and creates a shared trauma narrative; (2) core intervention using play and developmental guidance to process trauma narratives and manage in‐session distress; and (3) consolidation of gains and planning for future challenges.

CPP's efficacy is well documented through multiple randomized controlled trials demonstrating sustained decreases in PTSD and trauma‐related symptoms, improved parent–child attachment, and enhanced physiological regulation (Cicchetti, Rogosch, Toth, & Sturge‐Apple, 2011; Ghosh Ippen, Harris, Van Horn, & Lieberman, 2011; Lieberman, Ghosh Ippen, & Van Horn, 2006). In one key trial, though conducted with older children, 75 preschoolers exposed to intimate partner violence showed greater reductions in PTSD symptoms and behavior problems with CPP compared to case management with community referrals (Lieberman, Van Horn, & Ippen, 2005). Mothers in the CPP group also had reductions in PTSD symptoms, particularly avoidance symptoms. Sustained reductions in child behavior problems were observed 6 months after the trial. Importantly, children with exposure to multiple trauma types showed significant benefits (Ghosh Ippen et al., 2011).

For infants specifically, much of the empirical support examines attachment security and child emotional and behavioral problems rather than PTSD symptoms given the challenges with assessment in this age group. CPP represents the leading intervention for trauma‐exposed infants and toddlers because it (1) addresses the developmental reality that infants process trauma through relationships, (2) offers flexibility to respond to moment‐to‐moment needs of the dyad, (3) demonstrates improvements in trauma symptoms and foundational developmental outcomes, and (4) creates sustainable change that extends beyond the treatment period (Lieberman et al., 2015). Thus, CPP was designed for children ages 0–6 and represents a suitable intervention for preschoolers as well as infants and toddlers.

### Interventions for preschoolers

Much of the work advancing recognition and measurement of PTSD among preschoolers has been shepherded by Dr. Michael Scheeringa and colleagues. Before the creation of Scheeringa's Preschool PTSD Treatment intervention (Scheeringa, Weems, Cohen, Amaya‐Jackson, & Guthrie, 2011), only two trials had focused specifically on preschool‐aged children, both exclusively involving preschoolers who had experienced sexual abuse (Cohen & Mannarino, 1996; Deblinger, Stauffer, & Steer, 2001).

Cohen and Mannarino (1996) randomly assigned 67 children to 12 sessions of TF‐CBT or control (nondirective supportive therapy) and demonstrated improvements in total behavior problems and internalizing problems, though they did not measure PTSD symptoms. Deblinger et al. (2001) did measure PTSD and demonstrated reductions following 11 TF‐CBT group sessions for preschoolers exposed to sexual abuse, though children randomized to a supportive educational group saw similar reductions. Importantly, neither group was prompted to speak about their trauma experiences given their young ages, meaning there was no exposure element, which is now recognized as an essential intervention component.

*Preschool PTSD Treatment (PPT)* was developed in response to the need for evidence‐based interventions for preschoolers exposed to diverse trauma types beyond sexual abuse. Developed by Scheeringa and colleagues (treatment manual: Scheeringa, 2015). PPT adapted Trauma‐Focused Cognitive Behavioral Therapy for the preschool population.

PPT includes 12 manualized sessions covering psychoeducation about PTSD, recognition of feelings, coping skills training, graduated exposure to trauma reminders through three modalities accessible to preschoolers (drawings, imaginal, and in vivo), and safety planning. Caregivers are involved in the entire session for sessions 1–2 and 12 (the final session). In all other sessions, the caregiver observes the child's individual sessions with the therapist through a one‐way mirror, which is firmly established in the first two parent–child sessions.

Caregivers observe their child with the therapist to learn the content and techniques, increasing caregiver knowledge and attunement with the child's experience. They then spend the latter half of the session with the therapist, who helps them understand and interpret the child's responses and provides emotional support. The caregiver‐therapist session also addresses homework assignments to be practiced between sessions.

Prior to the published trial, Scheeringa and colleagues published two case reports that documented the early development of PPT with a child involved in a motor vehicle accident (age 4.5 years) and a child impacted by Hurricane Katrina (age 3.5 years) (Scheeringa et al., 2007). These case reports established that preschoolers can meaningfully cooperate in CBT‐based intervention components including exposure exercises, successfully utilize relaxation techniques, and benefit from treatment regardless of caregiver symptomatology so long as caregivers can follow the manualized exercises.

In the key randomized controlled trial, children aged 36–83 months who had experienced a life‐threatening event were randomly assigned to PPT (*n* = 40) or waitlist control (*n* = 24). The study purposefully recruited children with three types of trauma exposure: acute single blow trauma, repeated exposure to domestic violence, and exposure to Hurricane Katrina. Children in the intervention group showed significant improvements in PTSD symptoms compared to controls, with differences persisting at 6‐month follow‐up. Treatment effects were consistent across trauma types and included improvements in comorbid conditions such as depression, separation anxiety, and oppositional defiant disorder (Scheeringa et al., 2011).

Importantly, Scheeringa and colleagues also reported on the feasibility of therapeutic techniques by age. They demonstrated that children ages 3–6 were able to engage in most tasks, though 3‐year‐olds sometimes struggled with understanding concepts following verbal discussion, identifying feelings, and identifying distinct aspects of the trauma. However, therapists were largely able to make tasks more tangible through visual aids and drawings.

Although supported by only one randomized trial, PPT's strong theoretical grounding in TF‐CBT principles and careful age‐appropriate adaptations make it an exemplar for treating trauma in preschoolers. It remains the only intervention specifically designed for preschoolers that demonstrates efficacy across diverse trauma types.

### Interventions for early elementary children

Interventions for early elementary children exposed to trauma are more prevalent and thoroughly researched compared to earlier ages. Although it took time for the field to recognize children experience trauma and develop PTSD, there has been generally widespread acceptance that children ages 6–8 years experience trauma and benefit from treatment.

*Trauma‐Focused Cognitive Behavioral Therapy (TF‐CBT)* has the strongest evidence base for children ages 6–8 years. Pioneered by Drs. Judy Cohen, Esther Deblinger, and Anthony Mannarino, TF‐CBT has demonstrated efficacy across multiple randomized controlled trials with strong, sustained effects on PTSD symptoms (Cohen et al., 2017).

The TF‐CBT intervention model is organized into three phases: (1) safety/stabilization and skill‐building, where the clinician provides psychoeducation about trauma and treatments while helping the child build skills to cope with trauma reminders; (2) trauma narration and processing, where exploration of the trauma story allows for processing of thoughts and emotions while supporting gradual exposure; and (3) integration and consolidation, where the child works to gain mastery during in vivo exposure, practice skills, and plan for future safety.

A key focus of the final phase is enhancing parent–child communication about the trauma. In early phases, caregivers and children typically meet individually with the therapist, working on parallel components. Later phases incorporate joint parent–child sessions to enhance communication and reinforce skills.

The developers emphasize the importance of caregiver involvement, while noting that TF‐CBT is a child‐focused intervention. Caregivers with severe trauma‐related symptoms or serious psychosocial challenges are encouraged to seek their own individual treatment alongside participating with their child.

Multiple trials demonstrate TF‐CBT's efficacy for early elementary children. Much of the early work establishing efficacy focused on children who experienced sexual abuse. Later work examined the impact of treatment components and duration. Deblinger, Mannarino, Cohen, Runyon, and Steer (2011) randomly assigned 210 4–11‐year‐old children who experienced sexual abuse to one of four conditions: 8 weeks versus 16 weeks, with versus without trauma narrative. Reductions in PTSD symptoms were greater for those receiving 16 weeks of treatment, while the trauma narrative component was associated with reducing abuse‐related fear, general anxiety, and caregiver distress. These gains were maintained at 12‐month follow‐up (Mannarino, Cohen, Deblinger, Runyon, & Steer, 2012).

Multiple meta‐analyses confirm TF‐CBT's robust effects across different trauma types and diverse populations. The intervention has demonstrated effectiveness in reducing trauma symptoms, PTSD diagnoses, and associated behavioral problems across numerous randomized controlled trials with children ages 6–8 years. The inclusion of the trauma narrative component appears particularly important for this age group, as it directly addresses trauma‐related fear and anxiety while facilitating adaptive cognitive processing of the traumatic experience.

*Stepped Care TF‐CBT (SC‐TF‐CBT)* represents an innovative adaptation designed to increase accessibility by addressing barriers to traditional TF‐CBT including time commitment, cost, and transportation. SC‐TF‐CBT is a stepped model where response to the initial step determines progression to more intensive treatment (Salloum, Scheeringa, Cohen, & Storch, 2014).

Step 1 consists of caregiver‐led treatment completed at home, supported by 3 in‐office sessions with a therapist, phone support, and web‐based psychoeducational materials (Salloum et al., 2014). If children respond to Step 1, they enter a 6‐week maintenance phase with continued caregiver–child meetings and skill practice. Children requiring additional support enter Step 2, comprising 9 weeks of therapist‐led TF‐CBT sessions.

In a randomized trial comparing SC‐TF‐CBT to standard TF‐CBT for children aged 3–7 years, 71% of children responded to Step 1 alone. Both interventions were effective in reducing PTSD symptoms, with no differences between groups in rates of PTSD diagnoses at posttreatment or 3‐month follow‐up. Treatment costs were considerably lower for SC‐TF‐CBT (Salloum et al., 2016). Further analysis demonstrated that SC‐TF‐CBT is also non‐inferior for reducing caregiver posttraumatic stress and depression, though children with significant externalizing problems may benefit more from standard TF‐CBT (Salloum et al., 2022).

SC‐TF‐CBT represents a promising innovation that maintains clinical effectiveness while addressing accessibility barriers. It may be particularly indicated for families with transportation difficulties, those in rural areas with limited access to specialists, or caregivers with scheduling constraints. As implementation science continues to advance, SC‐TF‐CBT stands as an important step toward making evidence‐based trauma treatment more accessible without compromising effectiveness.

### Emerging interventions with limited support

Our review identified several emerging interventions with preliminary evidence that warrant attention. These approaches represent innovative methods for addressing trauma in young children and highlight important directions for future research.

*Coping with Accident Reactions (CARE)* is a preventive intervention for young children who experienced injuries and show risk factors for developing PTSD (De Young, Haag, Kenardy, Kimble, & Landolt, 2016). In a randomized controlled trial with children ages 1–6 years, CARE provided two 45–60 min sessions within 2 weeks of injury, focusing on psychoeducation about trauma reactions, coping strategies, and effective parenting responses (Haag et al., 2020).

The intervention is structured around two key sessions. The first session focuses on normalizing trauma reactions, teaching coping skills for both caregiver and child distress, and supporting caregivers in creating a coherent narrative about the accident and medical treatment. The second session, conducted 1 week later, addresses how parenting behaviors may change following traumatic injuries, helps caregivers identify unhelpful strategies such as overprotectiveness or excessive guilt, and develops goals for effective parenting responses. Follow‐up sessions check progress and provide additional referrals if needed.

While between‐group differences in PTSD symptoms were not statistically significant (*p* = .055) in the trial, promising trends were observed, including faster symptom improvement in the intervention group and the notable finding that no children in the intervention group (*n* = 26) met full PTSD criteria at 6‐month follow‐up compared to five children in the control group (*n* = 27). CARE demonstrates significant potential as a brief, targeted preventive intervention that could be feasibly implemented in medical settings following acute injuries.

*Neurofeedback training (NFT)* represents a neurobiologically focused approach targeting the physiological dysregulation underlying trauma symptoms (Huang‐Storms, Bodenhamer‐Davis, Davis, & Dunn, 2007). NFT teaches children to modify brain activity patterns associated with hyperarousal and hypervigilance by providing real‐time visual and/or auditory feedback about brain wave patterns. This approach theoretically addresses the neurobiological disruptions in emotion regulation, attention, and stress response systems that often follow early childhood trauma.

NFT for trauma typically focuses on normalizing patterns of cortical activity, particularly addressing hyperarousal by training children to enhance alpha wave activity (associated with relaxed alertness) while reducing theta wave activity (associated with drowsiness) and beta wave activity (associated with active thinking and potential anxiety). Through this process, NFT aims to help children develop improved physiological self‐regulation, potentially reducing intrusive thoughts, emotional reactivity, and hyperarousal symptoms.

One randomized trial with 37 trauma‐exposed children aged 6–13 years showed initial promise for reducing externalizing symptoms and improving behavioral regulation (Rogel et al., 2020). Twenty children received 24 twice‐weekly NFT sessions over 12 weeks, with assessments conducted at baseline, midpoint, endpoint, and 1 month after intervention completion. While there was initially a significant effect on rates of PTSD diagnoses at midpoint and endpoint assessments (relative to waitlist/control, *n* = 17), this difference was not maintained at the 1‐month follow‐up—suggesting potential issues with durability of effects.

The theoretical rationale for NFT is compelling, particularly given what we know about trauma's neurobiological impacts, but methodological limitations in existing research prevent strong conclusions about its efficacy. Future research should employ larger samples, longer follow‐up periods, and comparison to active treatment controls. Research should also investigate whether combining NFT with more traditional therapeutic approaches might produce more durable effects by addressing both neurobiological dysregulation and cognitive‐behavioral processes simultaneously.

*Eye Movement Integration (EMI)* has been proposed for young children based on theories about integrating fragmented trauma memories through directed eye movements (Struwig & Van Breda, 2012). Unlike Eye Movement Desensitization and Reprocessing (EMDR), which uses horizontal rapid eye movements, EMI uses smooth pursuit eye movements following specific patterns across multiple directions. While EMDR has shown efficacy with older children and adolescents, published trials with children under age 8 are limited, precluding inclusion in this developmentally focused review. The theoretical premise for EMI and EMDR is that eye movements facilitate integration of sensory, emotional, and cognitive aspects of traumatic memories, particularly for children who may not have the verbal capacities to process trauma through traditional cognitive means.

There has only been one study on EMI for young children, which reported a reduction in PTSD symptoms as well as depression, anxiety, and anger immediately following a single EMI session with 12 children aged 5–7 years (Van Der Spuy & van Breda, 2019). However, this study had substantial methodological limitations, including a small convenience sample, lack of control group, absence of follow‐up assessments, and potential researcher bias (the first author provided the treatment and led the study). Furthermore, the involvement of caregivers in the intervention was not clearly described, which is concerning given the crucial role of caregivers in trauma treatment for young children.

Given these substantial limitations and the single‐session nature of the intervention, EMI should be considered highly preliminary for young children exposed to trauma. The current evidence base is insufficient to recommend clinical implementation. Future research would need to address the methodological limitations noted above, establish a clearer protocol for caregiver involvement, and demonstrate both safety and sustained efficacy.

Together, these emerging interventions highlight the field's efforts to diversify treatment approaches for trauma‐exposed young children. CARE shows the most promise with its preventive approach and strong methodological foundation, while NFT raises intriguing possibilities for addressing neurobiological aspects of trauma that may not be fully addressed by traditional psychotherapeutic interventions. EMI represents an approach that theoretically addresses the challenges of treating trauma in children with limited verbal abilities, though its evidence base requires substantial strengthening. While clinicians should prioritize established evidence‐based interventions, these emerging approaches warrant continued research attention to determine whether they might address current gaps in trauma treatment for young children.

## Innovations and future directions

### Alternative approaches: Caregiver‐focused interventions

An important trend in the literature is the development of interventions for caregivers of trauma‐exposed young children, particularly following medical traumas. These approaches recognize that supporting caregivers' well‐being enhances their ability to buffer children from trauma effects.

Several studies have evaluated interventions for caregivers with trauma symptoms stemming from their child's medical experiences. For example, Sveen, Andersson, Buhrman, Sjöberg, and Willebrand (2017) tested an internet‐based support program for caregivers of children with burns, finding reductions in caregivers' posttraumatic stress symptoms. Similarly, Muscara et al. (2020) demonstrated the efficacy of Take a Breath, an online group intervention based on acceptance and commitment therapy for caregivers of children with life‐threatening illnesses.

Such caregiver‐focused interventions may indirectly benefit children by improving caregiver functioning. One study of caregivers whose children experienced unexpected hospitalization reported not only caregiver mental health improvements but also significant reductions in child behavior problems following intervention (Melnyk et al., 2004).

### Expanding the evidence base for non‐interpersonal trauma

Interpersonal traumas, particularly child maltreatment, dominate the intervention literature for young children. Exposure to non‐interpersonal traumatic events (e.g., accidents, natural disasters, medical procedures) has received less attention despite potentially profound impacts.

One promising direction is the development of preventive interventions following potentially traumatic medical events. Preliminary evidence suggests that early, brief interventions may prevent chronic PTSD following injuries or acute medical events (Kassam‐Adams et al., 2016). Similarly, adaptations of established treatments like CPP for medical contexts, such as for caregivers with infants in the NICU (Lakatos, Matic, Carson, & Williams, 2019), represent important innovations.

Research on interventions following natural disasters represents another significant gap. Despite evidence that natural disasters affect millions of children annually, few studies examine interventions specifically for young children exposed to these events. Existing evidence suggests that CBT‐based approaches effective for other trauma types may also benefit disaster‐exposed children (Burkhart et al., 2023; Forman‐Hoffman et al., 2013; Le Roux & Cobham, 2022) but more direct research is needed.

### Assessment and diagnosis challenges

One of the more prominent challenges noted across the literature on trauma during early childhood is identifying the presence of PTSD in young children, which can be a barrier to identification and referral to intervention. Historically, PTSD was often under‐ or misdiagnosed in young children due to differences in its clinical presentation compared to older children and—until relatively recently—a lack of advancements in assessment and diagnosis (Cohen, Mannarino, & Rogal, 2001). There is also widespread misinformation about the impact of trauma on young children and the fact that even very young infants can exhibit trauma symptoms is a more recent line of study (Chu et al., 2025). It is important to note that a formal PTSD diagnosis is not required to initiate trauma‐focused treatment. A symptom‐based, trauma‐informed approach that treats distress and functional impairment following identified trauma exposure aligns with trauma‐informed care principles and reflects real‐world clinical practice.

The assessment of PTSD in young children is typically viewed as more complicated than other common disorders due to the fundamental nature of PTSD symptoms needing to be directly linked to an identifiable trauma exposure. Clinicians must recognize connections between child behavior and traumatic experiences, which requires careful observation by both clinicians and caregivers since children cannot reliably report on their own internal states (Gleason et al., 2007). It can be challenging to determine when child behavior represents a reaction to a trauma trigger because adults are not privy to the universe of trauma reminders that are salient to the child and must rely on assumptions about the child's experience.

The field has come a long way in the assessment and diagnosis of PTSD in young children with the development and refinement of assessment tools and enhanced diagnostic criteria. Following calls for changes to the DSM‐IV criteria to better accommodate the presentations of young children (De Young, Kenardy, & Cobham, 2011; Scheeringa, Myers, Putnam, & Zeanah, 2012), DSM‐5 (APA, 2013) debuted in 2013 with new PTSD diagnostic guidelines for preschool‐aged children. The diagnosis of PTSD among infants and toddlers is best captured through the DC: 0–5 diagnostic classification system (Zero to Three, 2016), a specialized tool for children ages 0–5 years that includes the Disorder of Traumatic Stress diagnosis.

For infants and toddlers (0–3 years), assessment relies heavily on observational measures and caregiver report. Instruments such as the Infant‐Toddler Social Emotional Assessment (ITSEA; Carter, Briggs‐Gowan, Jones, & Little, 2003) can capture behavioral manifestations of trauma that may not be verbally expressed. Clinicians must be skilled at recognizing trauma‐specific symptoms that may present as developmental regressions, changes in attachment behaviors, disrupted sleep, heightened startle responses, and repetitive play themes related to the traumatic event.

For preschoolers (3–5 years), the most well‐validated structured clinical interviews are the Diagnostic Infant and Preschool Assessment (DIPA; Scheeringa, 2020; Scheeringa & Haslett, 2010) and the Preschool Age Psychiatric Assessment (PAPA; Egger et al., 2006). Both demonstrate good psychometric properties and include developmentally modified PTSD criteria. Another option for children under 6 is the UCLA PTSD Reaction Index for DSM‐5 (Steinberg, Pynoos, Lieberman, Osofsky, & Vivrette, 2017). These can be supplemented with caregiver‐report measures such as the Young Child PTSD Checklist (YCPC; Scheeringa, 2013) and the Trauma Symptom Checklist for Young Children (TSCYC; Briere et al., 2001; Pollio, Glover‐Orr, & Wherry, 2008; Wherry, Graves, & Rhodes King, 2008), which provide efficient screening tools. In this age group, children may begin to verbalize some trauma‐related thoughts and feelings, though concrete thinking limits their ability to connect symptoms to traumatic events without adult assistance.

For early elementary children (6–8 years), clinicians can begin to integrate child self‐report alongside caregiver reports. The Trauma Symptom Checklist for Children (TSCC; Briere, 1996) has a version appropriate for children as young as 8, while the Child PTSD Symptom Scale (CPSS; Foa, Johnson, Feeny, & Treadwell, 2001) has been used successfully with children as young as 7. For structured interviews, the Clinician‐Administered PTSD Scale for Children and Adolescents (CAPS‐CA; Nader et al., 1996) and the Schedule for Affective Disorders and Schizophrenia for School‐Age Children (K‐SADS; Kaufman et al., 1997) provide comprehensive assessment options.

A particular challenge is the longitudinal assessment of trauma symptoms across development. As children mature from infancy through early elementary years, symptom manifestations change dramatically. To illustrate this developmental progression, consider how reexperiencing symptoms might manifest and be measured in a child who experienced a serious car accident at age 2:

- During toddlerhood, this symptom might appear as night terrors, distress when riding in cars, and repetitive play involving toy cars crashing. Assessment would rely entirely on caregiver observation and report
- By age 4 years, the same child might verbalize frightening dreams about cars or monsters, engage in more elaborate accident‐related play, and answer simple questions about feelings when seeing cars or hearing loud noises. Assessment could now incorporate simple self‐report alongside caregiver observations.
- By age 7 years, this child could potentially describe intrusive thoughts about the accident, articulate feelings of fear when remembering it, and make connections between current triggers and the original trauma. Assessment could now include standardized self‐report measures and direct questions about thought content.

This developmental progression creates significant challenges for longitudinal assessment. A measure appropriate at age 2 years would be insufficiently comprehensive by age 7 years, while measures designed for older children would be inappropriate for toddlers. Even when using age‐appropriate measures at each time point, distinguishing genuine symptom changes from developmental changes in expression and measurement presents a formidable methodological challenge. Researchers must carefully consider whether observed symptom reductions represent true treatment effects or simply the child's advancing cognitive and verbal abilities allowing them to better regulate and integrate traumatic experiences as part of normal development.

Despite these challenges, the field has made substantial progress in developing valid and reliable assessment tools for each developmental stage. Multi‐method, multi‐informant assessment approaches yield the most comprehensive understanding of a child's trauma symptoms and should be considered best practice. Future research should focus on developing assessment approaches that can track trauma symptoms longitudinally across developmental transitions while accounting for normative developmental changes in symptom expression.

### Age considerations for treatment selection

The optimal intervention approach for children ages 6–8 years remains an important empirical question. These children sit at the intersection between interventions designed specifically for young children and those developed for broader child and adolescent populations. Meta‐analytic findings on interventions for children and adolescents with anxiety disorders, including PTSD, have reported smaller effects of CBT‐based interventions for children under age 13 years compared to adolescents (Reynolds, Wilson, Austin, & Hooper, 2012). However, another meta‐analysis found higher efficacy when interventions had a component of caregiver involvement (Gutermann et al., 2016). Most meta‐analyses include few studies with children under 8, making definitive conclusions difficult for this specific age group. For this transitional age group, several factors beyond chronological age should guide intervention selection.

The first consideration is the child's developmental maturity. School‐age children show significant variability in cognitive, verbal, emotional, and social development. A 6‐year‐old with advanced verbal abilities and abstract thinking may benefit from more cognitively oriented approaches typically used with older children, whereas an 8‐year‐old with developmental delays or concrete thinking may respond better to play‐based approaches designed for younger children. Assessment of developmental functioning across multiple domains should guide treatment planning more than chronological age alone.

Verbal abilities deserve particular attention, as they directly impact a child's capacity to engage in various therapeutic modalities. Children with strong expressive and receptive language skills can benefit from interventions that incorporate verbal processing of traumatic experiences, including the creation of trauma narratives. Conversely, children with language limitations may require more reliance on nonverbal techniques such as play, art, or embodied activities.

The quality of the caregiver–child relationship represents another crucial factor. When the relationship is secure and the caregiver is emotionally regulated and capable of supporting the child's recovery, interventions with caregiver involvement may yield the strongest outcomes. This is particularly true when trauma occurred outside the caregiving relationship, allowing the caregiver to serve as a secure base for exploration of difficult emotions. However, when caregivers are themselves highly traumatized, emotionally dysregulated, or contribute to the child's trauma, a different approach may be needed. In these cases, parallel services for caregivers alongside child‐focused intervention may be indicated.

Child preferences should also be considered, particularly as children approach school age and develop increased autonomy and self‐awareness. Some children actively seek opportunities to talk about their experiences and may engage readily in verbal processing, while others prefer expressive modalities like play or art. Honoring these preferences, when clinically appropriate, can enhance engagement and treatment acceptability.

Practical constraints inevitably influence treatment selection as well. Service availability, insurance coverage, transportation challenges, and caregiver work schedules may limit which interventions are realistically accessible. While evidence‐based practices should guide recommendations whenever possible, clinicians must balance empirical support with feasibility. A theoretically “second‐best” intervention that can be consistently implemented may yield better outcomes than a “gold standard” approach that faces significant barriers to completion.

**Figure 1:**
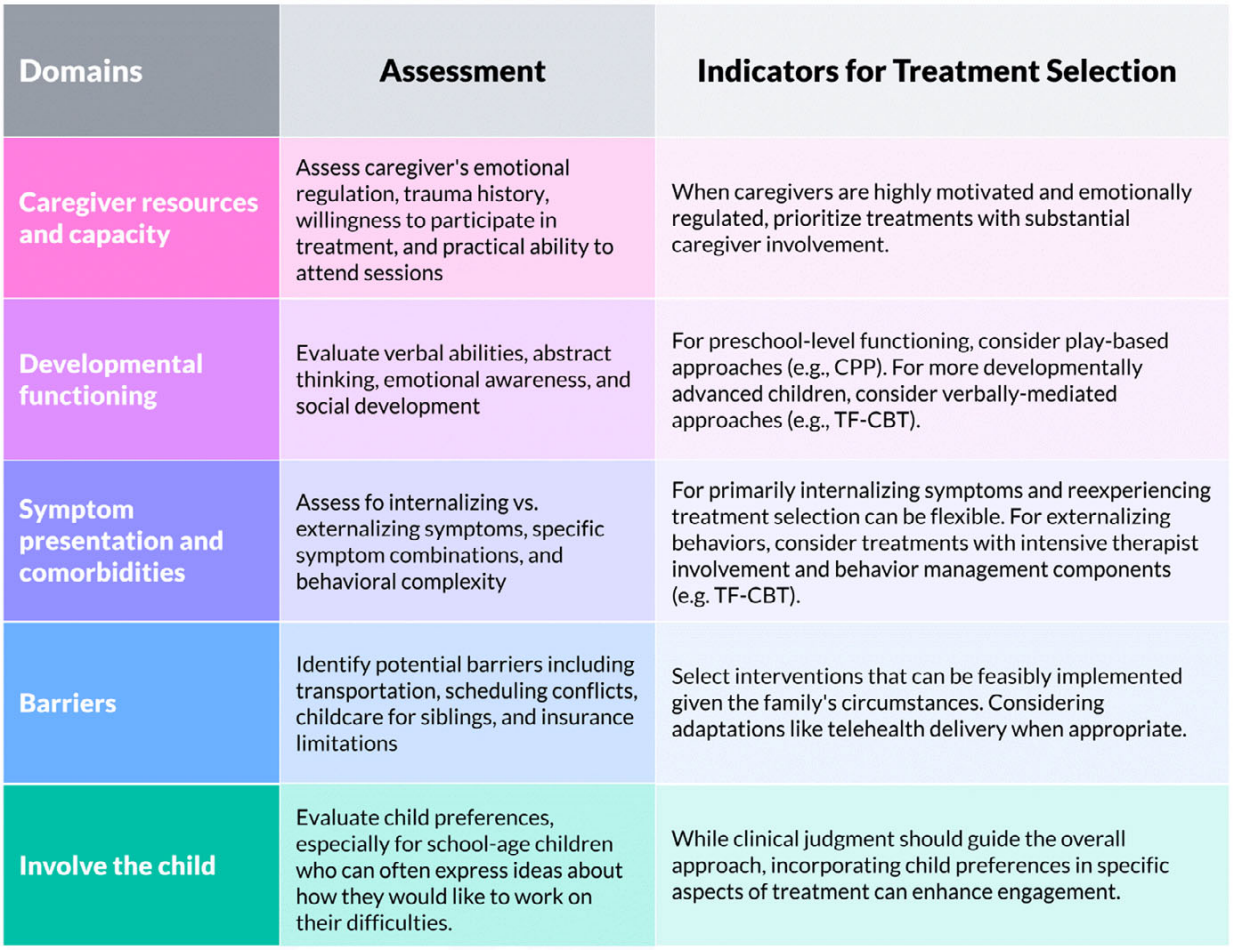
Decision‐making framework for treatment selection in trauma‐exposed children ages 6–8 years. CPP, Child–Parent Psychotherapy; TF‐CBT, trauma‐focused CBT

Based on our review of the literature and clinical experience, we propose a decision‐making framework to guide clinicians working with trauma‐exposed children ages 6–8 years (Figure 1).

When implementing this framework, it is important to recognize that many 6–8 year olds will fall into a “hybrid” zone where they show some characteristics suggesting readiness for more verbal approaches alongside continued needs for play‐based, caregiver‐mediated intervention. In these cases, clinicians may need to flexibly integrate elements from multiple evidence‐based approaches, drawing principles from both younger and older child interventions. This developmental bridging approach may be particularly valuable for addressing the unique needs of this transitional age group.

## Conclusion

The landscape of trauma‐focused interventions for young children has evolved significantly, though substantial gaps remain. Our review reveals a tiered evidence base across the 0–8 age range, with the strongest support for interventions targeting specific age groups: Child–Parent Psychotherapy for infants and toddlers, Preschool PTSD Treatment for preschoolers, and Trauma‐Focused CBT for early elementary children.

Developmental considerations fundamentally shape trauma intervention for young children. The rapid neurodevelopmental changes across early childhood necessitate interventions specifically designed for children's evolving capacities rather than simply “scaled down” versions of adult treatments. For the youngest children, interventions operate primarily through the caregiver–child relationship, with the degree of direct child engagement increasing with age.

Critical gaps in current knowledge include (1) limited interventions addressing trauma in infants under age 3; (2) sparse evidence for noninterpersonal trauma interventions; (3) assessment challenges, particularly for longitudinal measurement across developmental transitions; and (4) insufficient implementation research on disseminating specialized interventions in community settings.

Future research priorities should include (1) expanding the evidence base for existing promising interventions through well‐powered trials with diverse samples, (2) developing and testing preventive interventions delivered following potentially traumatic events, (3) adapting established interventions for under‐studied trauma types, and (4) implementation research to support widespread adoption in real‐world settings.

The stakes for advancing this field could not be higher. Early childhood trauma exposure occurs during sensitive periods of brain development, with potential lifelong consequences. Yet this same developmental plasticity creates unique opportunities for intervention to redirect trajectories toward positive outcomes. By continuing to refine effective trauma interventions for our youngest children, we can not only alleviate immediate suffering but potentially prevent decades of associated difficulties across the lifespan.

## Ethical considerations

Not applicable. No data from human subjects.

**Key points**

**What is known?**

- The landscape of trauma‐focused interventions for young children has evolved significantly, though substantial gaps remain.
- Developmental considerations, such as cognitive development and verbal abilities, fundamentally shape trauma intervention for young children.

**What is new?**

- For the youngest children, interventions operate primarily through the caregiver–child relationship, with the degree of direct child engagement increasing with age.
- Critical gaps in current knowledge include (1) limited interventions addressing trauma in infants under age 3; (2) sparse evidence for noninterpersonal trauma interventions; (3) assessment challenges, particularly for longitudinal measurement across developmental transitions; and (4) insufficient implementation research on disseminating specialized interventions in community settings.

**What is relevant?**

- Future research priorities should include (1) expanding the evidence base for existing promising interventions through well‐powered trials with diverse samples; (2) developing and testing preventive interventions delivered following potentially traumatic events; (3) adapting established interventions for under‐studied trauma types; and (4) implementation research to support widespread adoption in real‐world settings.
- By continuing to refine effective trauma interventions for our youngest children, we can not only alleviate immediate suffering but also potentially prevent decades of associated difficulties across the lifespan.

## Data Availability

Data sharing is not applicable to this article as no datasets were generated or analyzed during the current study.

## Endnote

1 Complex trauma refers to exposure to multiple traumatic events often occurring during childhood and the wide‐ranging impacts of such exposure.

## References

[jcpp70121-bib-0001] American Psychiatric Association . (2013). Diagnostic and statistical manual of mental disorders (5th edn). Arlington, VA: American Psychiatric Publishing.

[jcpp70121-bib-0002] Andersen, S.L. , & Teicher, M.H. (2008). Stress, sensitive periods and maturational events in adolescent depression. Trends in Neurosciences, 31, 183–191. 18329735 10.1016/j.tins.2008.01.004

[jcpp70121-bib-0003] Briere, J. (1996). Trauma symptom checklist for children: Professional manual. Odessa, FL: Psychological Assessment Resources.

[jcpp70121-bib-0004] Briere, J. , Johnson, K. , Bissada, A. , Damon, L. , Crouch, J. , Gil, E. , … & Ernst, V. (2001). The Trauma Symptom Checklist for Young Children (TSCYC): Reliability and association with abuse exposure in a multi‐site study. Child Abuse & Neglect, 25, 1001–1014. 11601594 10.1016/s0145-2134(01)00253-8

[jcpp70121-bib-0005] Burkhart, K. , Agarwal, N. , Kim, S. , Neudecker, M. , & Ievers‐Landis, C.E. (2023). A scoping review of trauma‐informed pediatric interventions in response to natural and biologic disasters. Children, 10, 1017. 37371249 10.3390/children10061017 PMC10297269

[jcpp70121-bib-0006] Carter, A.S. , Briggs‐Gowan, M.J. , Jones, S.M. , & Little, T.D. (2003). The Infant‐Toddler Social and Emotional Assessment (ITSEA): Factor structure, reliability, and validity. Journal of Abnormal Child Psychology, 31, 495–514. 14561058 10.1023/a:1025449031360

[jcpp70121-bib-0008] Chu, A.T. , Bond, M.H. , Rogowski, B. , Leba, N.V. , Ghosh Ippen, C. , Cirolia, A. , & Lieberman, A.F. (2025). Posttraumatic stress in infancy: The roles of cumulative trauma and caregiving context. Infant Mental Health Journal, 46, 536–548. 40275536 10.1002/imhj.70015

[jcpp70121-bib-0009] Cicchetti, D. , Rogosch, F.A. , Toth, S.L. , & Sturge‐Apple, M.L. (2011). Normalizing the development of cortisol regulation in maltreated infants through preventive interventions. Development and Psychopathology, 23, 789–800. 21756432 10.1017/S0954579411000307 PMC4191893

[jcpp70121-bib-0010] Cicchetti, D. , & Toth, S.L. (2016). Child maltreatment and developmental psychopathology: A multilevel perspective. In D. Cicchetti (Ed.), Developmental psychopathology: Maladaptation and psychopathology (pp. 457–512). Hoboken, NJ: John Wiley & Sons.

[jcpp70121-bib-0012] Cohen, J.A. , & Mannarino, A.P. (1996). A treatment outcome study for sexually abused preschool children: Initial findings. Journal of the American Academy of Child and Adolescent Psychiatry, 35, 42–50. 8567611 10.1097/00004583-199601000-00011

[jcpp70121-bib-0014] Cohen, J.A. , Mannarino, A.P. , & Deblinger, E. (2017). Treating trauma and traumatic grief in children and adolescents (2nd edn). New York, NY: Guilford Press.

[jcpp70121-bib-0015] Cohen, J.A. , Mannarino, A.P. , & Rogal, S. (2001). Treatment practices for childhood posttraumatic stress disorder. Child Abuse & Neglect, 25, 123–135. 11214806 10.1016/s0145-2134(00)00226-x

[jcpp70121-bib-0016] Cook, A. , Spinazzola, J. , Ford, J. , Lanktree, C. , Blaustein, M. , Cloitre, M. , … & van der Kolk, B. (2005). Complex trauma in children and adolescents. Psychiatric Annals, 35, 390–398.

[jcpp70121-bib-0017] De Arellano, M.a.R. , Lyman, D.R. , Jobe‐Shields, L. , George, P. , Dougherty, R.H. , Daniels, A.S. , … & Delphin‐Rittmon, M.E. (2014). Trauma‐focused cognitive‐behavioral therapy for children and adolescents: Assessing the evidence. Psychiatric Services, 65, 591–602. 24638076 10.1176/appi.ps.201300255 PMC4396183

[jcpp70121-bib-0018] De Young, A.C. , Haag, A.‐C. , Kenardy, J.A. , Kimble, R.M. , & Landolt, M.A. (2016). Coping with accident reactions (CARE) early intervention programme for preventing traumatic stress reactions in young injured children: Study protocol for two randomised controlled trials. Trials, 17, 1–10. 27464735 10.1186/s13063-016-1490-2 PMC4964020

[jcpp70121-bib-0019] De Young, A.C. , Kenardy, J.A. , & Cobham, V.E. (2011). Diagnosis of posttraumatic stress disorder in preschool children. Journal of Clinical Child & Adolescent Psychology, 40, 375–384. 21534049 10.1080/15374416.2011.563474

[jcpp70121-bib-0021] Deblinger, E. , Mannarino, A.P. , Cohen, J.A. , Runyon, M.K. , & Steer, R.A. (2011). Trauma‐focused cognitive behavioral therapy for children: Impact of the trauma narrative and treatment length. Depression and Anxiety, 28, 67–75. 20830695 10.1002/da.20744 PMC6675414

[jcpp70121-bib-0022] Deblinger, E. , Stauffer, L.B. , & Steer, R.A. (2001). Comparative efficacies of supportive and cognitive behavioral group therapies for young children who have been sexually abused and their nonoffending mothers. Child Maltreatment, 6, 332–343. 11675816 10.1177/1077559501006004006

[jcpp70121-bib-0023] Egger, H.L. , Erkanli, A. , Keeler, G. , Potts, E. , Walter, B.K. , & Angold, A. (2006). Test‐retest reliability of the preschool age psychiatric assessment (PAPA). Journal of the American Academy of Child and Adolescent Psychiatry, 45, 538–549. 16601400 10.1097/01.chi.0000205705.71194.b8

[jcpp70121-bib-0024] Feldman, R. , & Vengrober, A. (2011). Posttraumatic stress disorder in infants and young children exposed to war‐related trauma. Journal of the American Academy of Child & Adolescent Psychiatry, 50, 645–658. 21703492 10.1016/j.jaac.2011.03.001

[jcpp70121-bib-0025] Foa, E.B. , Johnson, K.M. , Feeny, N.C. , & Treadwell, K.R. (2001). The child PTSD symptom scale: A preliminary examination of its psychometric properties. Journal of Clinical Child Psychology, 30, 376–384. 11501254 10.1207/S15374424JCCP3003_9

[jcpp70121-bib-0026] Forman‐Hoffman, V.L. , Zolotor, A.J. , McKeeman, J.L. , Blanco, R. , Knauer, S.R. , Lloyd, S.W. , … & Viswanathan, M. (2013). Comparative effectiveness of interventions for children exposed to nonrelational traumatic events. Pediatrics, 131, 526–539. 23400617 10.1542/peds.2012-3846

[jcpp70121-bib-0027] Ghosh Ippen, C. , Harris, W.W. , Van Horn, P. , & Lieberman, A.F. (2011). Traumatic and stressful events in early childhood: Can treatment help those at highest risk? Child Abuse and Neglect, 35, 504–513. 21816474 10.1016/j.chiabu.2011.03.009 PMC3159839

[jcpp70121-bib-0028] Gleason, M.M. , Egger, H.L. , Emslie, G.J. , Greenhill, L.L. , Kowatch, R.A. , Lieberman, A.F. , … & Zeanah, C.H. (2007). Psychopharmacological treatment for very young children: Contexts and guidelines. Journal of the American Academy of Child & Adolescent Psychiatry, 46, 1532–1572. 18030077 10.1097/chi.0b013e3181570d9e

[jcpp70121-bib-0029] Gunnar, M.R. , & Quevedo, K. (2007). The neurobiology of stress and development. Annual Review of Psychology, 58, 145–173. 10.1146/annurev.psych.58.110405.085605 16903808

[jcpp70121-bib-0030] Gutermann, J. , Schreiber, F. , Matulis, S. , Schwartzkopff, L. , Deppe, J. , & Steil, R. (2016). Psychological treatments for symptoms of posttraumatic stress disorder in children, adolescents, and young adults: A meta‐analysis. Clinical Child and Family Psychology Review, 19, 77–93. 27059619 10.1007/s10567-016-0202-5

[jcpp70121-bib-0031] Haag, A.‐C. , Landolt, M.A. , Kenardy, J.A. , Schiestl, C.M. , Kimble, R.M. , & De Young, A.C. (2020). Preventive intervention for trauma reactions in young injured children: Results of a multi‐site randomised controlled trial. Journal of Child Psychology and Psychiatry, and Allied Disciplines, 61, 988–997. 31912485 10.1111/jcpp.13193

[jcpp70121-bib-0032] Huang‐Storms, L. , Bodenhamer‐Davis, E. , Davis, R. , & Dunn, J. (2007). QEEG‐guided neurofeedback for children with histories of abuse and neglect: Neurodevelopmental rationale and pilot study. Journal of Neurotherapy, 10, 3–16.

[jcpp70121-bib-0034] Janoff‐Bulman, R. (1992). Shattered assumptions: Towards a new psychology of trauma. New York, NY: Free Press.

[jcpp70121-bib-0035] Kaplow, J.B. , & Widom, C.S. (2007). Age of onset of child maltreatment predicts long‐term mental health outcomes. Journal of Abnormal Psychology, 116, 176–187. 17324028 10.1037/0021-843X.116.1.176

[jcpp70121-bib-0036] Kassam‐Adams, N. , Marsac, M.L. , Kohser, K.L. , Kenardy, J. , March, S. , & Winston, F.K. (2016). Pilot randomized controlled trial of a novel web‐based intervention to prevent posttraumatic stress in children following medical events. Journal of Pediatric Psychology, 41, 138–148. 26089554 10.1093/jpepsy/jsv057 PMC4723670

[jcpp70121-bib-0037] Kaufman, J. , Birmaher, B. , Brent, D. , Rao, U. , Flynn, C. , Moreci, P. , … & Ryan, N. (1997). Schedule for Affective Disorders and Schizophrenia for school‐age children‐present and lifetime version (K‐SADS‐PL): Initial reliability and validity data. Journal of the American Academy of Child & Adolescent Psychiatry, 36, 980–988. 9204677 10.1097/00004583-199707000-00021

[jcpp70121-bib-0038] Lakatos, P.P. , Matic, T. , Carson, M. , & Williams, M.E. (2019). Child‐parent psychotherapy with infants hospitalized in the neonatal intensive care unit. Journal of Clinical Psychology in Medical Settings, 26, 584–596. 30941622 10.1007/s10880-019-09614-6

[jcpp70121-bib-0039] Le Roux, I.H. , & Cobham, V.E. (2022). Psychological interventions for children experiencing PTSD after exposure to a natural disaster: A scoping review. Clinical Child and Family Psychology Review, 25, 249–282. 34779953 10.1007/s10567-021-00373-1

[jcpp70121-bib-0040] Lieberman, A.F. , Ghosh Ippen, C. , & Van Horn, P. (2006). Child‐parent psychotherapy: 6‐month follow‐up of a randomized controlled trial. Journal of the American Academy of Child and Adolescent Psychiatry, 45, 913–918. 16865033 10.1097/01.chi.0000222784.03735.92

[jcpp70121-bib-0041] Lieberman, A.F. , Ghosh Ippen, C. , & Van Horn, P. (2015). Don't hit my mommy!: A manual for child‐parent psychotherapy with young witnesses of family violence (2nd edn). Washington DC: Zero to Three.

[jcpp70121-bib-0043] Lieberman, A.F. , Van Horn, P. , & Ippen, C.G. (2005). Toward evidence‐based treatment: Child‐parent psychotherapy with preschoolers exposed to marital violence. Journal of the American Academy of Child and Adolescent Psychiatry, 44, 1241–1248. 16292115 10.1097/01.chi.0000181047.59702.58

[jcpp70121-bib-0044] Main, M. , & Solomon, J. (1990). Procedures for identifying infants as disorganized/disoriented during the Ainsworth strange situation. In M.T. Greenberg , D. Cicchetti , & E.M. Cummings (Eds.), Attachment in the preschool years: Theory, research, and intervention (pp. 121–160). Chicago, IL: University of Chicago Press.

[jcpp70121-bib-0045] Mannarino, A.P. , Cohen, J.A. , Deblinger, E. , Runyon, M.K. , & Steer, R.A. (2012). Trauma‐focused cognitive‐behavioral therapy for children: Sustained impact of treatment 6 and 12 months later. Child Maltreatment, 17, 231–241. 22763575 10.1177/1077559512451787 PMC8787755

[jcpp70121-bib-0046] McKinnon, A. , Scheeringa, M.S. , Meiser‐Stedman, R. , Watson, P. , De Young, A. , & Dalgleish, T. (2019). The dimensionality of proposed DSM‐5 PTSD symptoms in trauma‐exposed young children. Journal of Abnormal Child Psychology, 47, 1799–1809. 31172404 10.1007/s10802-019-00561-2 PMC6805819

[jcpp70121-bib-0047] Meiser‐Stedman, R. (2002). Towards a cognitive‐behavioral model of PTSD in children and adolescents. Clinical Child and Family Psychology Review, 5, 217–232. 12495267 10.1023/a:1020982122107

[jcpp70121-bib-0048] Melnyk, B.M. , Alpert‐Gillis, L. , Feinstein, N.F. , Crean, H.F. , Johnson, J. , Fairbanks, E. , … & Corbo‐Richert, B. (2004). Creating opportunities for parent empowerment: Program effects on the mental health/coping outcomes of critically ill young children and their mothers. Pediatrics, 113, e597–e607. 15173543 10.1542/peds.113.6.e597

[jcpp70121-bib-0050] Muscara, F. , McCarthy, M.C. , Rayner, M. , Nicholson, J.M. , Dimovski, A. , McMillan, L. , … & Walser, R. (2020). Effect of a videoconference‐based online group intervention for traumatic stress in parents of children with life‐threatening illness: A randomized clinical trial. JAMA Network Open, 3, e208507‐e208507. 32735335 10.1001/jamanetworkopen.2020.8507 PMC7395233

[jcpp70121-bib-0051] Nader, K. , Kriegler, J.A. , Blake, D.D. , Pynoos, R.S. , Newman, E. , & Weathers, F.W. (1996). Clinician‐administered PTSD scale, child and adolescent version. White River Junction, VT: National Center for PTSD.

[jcpp70121-bib-0053] Perry, B.D. , Pollard, R.A. , Blakley, T.L. , Baker, W.L. , & Vigilante, D. (1995). Childhood trauma, the neurobiology of adaptation, and “use‐dependent” development of the brain: How “states” become “traits”. Infant Mental Health Journal, 16, 271–291.

[jcpp70121-bib-0054] Pollio, E.S. , Glover‐Orr, L.E. , & Wherry, J.N. (2008). Assessing posttraumatic stress disorder using the trauma symptom checklist for young children. Journal of Child Sexual Abuse, 17, 89–100. 19842320 10.1080/10538710701884557

[jcpp70121-bib-0055] Reynolds, S. , Wilson, C. , Austin, J. , & Hooper, L. (2012). Effects of psychotherapy for anxiety in children and adolescents: A meta‐analytic review. Clinical Psychology Review, 32, 251–262. 22459788 10.1016/j.cpr.2012.01.005

[jcpp70121-bib-0056] Rogel, A. , Loomis, A.M. , Hamlin, E. , Hodgdon, H. , Spinazzola, J. , & van der Kolk, B. (2020). The impact of neurofeedback training on children with developmental trauma: A randomized controlled study. Psychological Trauma Theory Research Practice and Policy, 12, 918. 32658503 10.1037/tra0000648

[jcpp70121-bib-0057] Salloum, A. , Lu, Y. , Chen, H. , Salomon, K. , Scheeringa, M.S. , Cohen, J.A. , … & Storch, E.A. (2022). Child and parent secondary outcomes in stepped care versus standard care treatment for childhood trauma. Journal of Affective Disorders, 307, 87–96. 35331823 10.1016/j.jad.2022.03.049 PMC9035131

[jcpp70121-bib-0058] Salloum, A. , Scheeringa, M.S. , Cohen, J.A. , & Storch, E.A. (2014). Development of stepped care trauma‐focused cognitive‐behavioral therapy for young children. Cognitive and Behavioral Practice, 21, 97–108. 25411544 10.1016/j.cbpra.2013.07.004 PMC4233143

[jcpp70121-bib-0059] Salloum, A. , Wang, W. , Robst, J. , Murphy, T.K. , Scheeringa, M.S. , Cohen, J.A. , & Storch, E.A. (2016). Stepped care versus standard trauma‐focused cognitive behavioral therapy for young children. Journal of Child Psychology and Psychiatry, 57, 614–622. 26443493 10.1111/jcpp.12471 PMC4824681

[jcpp70121-bib-0060] Salmon, K. , & Bryant, R.A. (2002). Posttraumatic stress disorder in children: The influence of developmental factors. Clinical Psychology Review, 22, 163–188. 11806018 10.1016/s0272-7358(01)00086-1

[jcpp70121-bib-0061] Scheeringa, M.S. (2013). Young Child PTSD Checklist. New Orleans, LA: Tulane University School of Medicine

[jcpp70121-bib-0062] Scheeringa, M.S. (2015). Treating PTSD in preschoolers: A clinical guide. New York, NY: The Guilford Press.

[jcpp70121-bib-0063] Scheeringa, M.S. (2020). The diagnostic infant preschool assessment‐Likert version: Preparation, concurrent construct validation, and test–retest reliability. Journal of Child and Adolescent Psychopharmacology, 30, 326–334. 32159386 10.1089/cap.2019.0168

[jcpp70121-bib-0064] Scheeringa, M.S. , & Haslett, N. (2010). The reliability and criterion validity of the diagnostic infant and preschool assessment: A new diagnostic instrument for young children. Child Psychiatry & Human Development, 41, 299–312. 20052532 10.1007/s10578-009-0169-2 PMC2862973

[jcpp70121-bib-0065] Scheeringa, M.S. , Myers, L. , Putnam, F.W. , & Zeanah, C.H. (2012). Diagnosing PTSD in early childhood: An empirical assessment of four approaches. Journal of Traumatic Stress, 25, 359–367. 22806831 10.1002/jts.21723 PMC6080618

[jcpp70121-bib-0066] Scheeringa, M.S. , Salloum, A. , Arnberger, R.A. , Weems, C.F. , Amaya‐Jackson, L. , & Cohen, J.A. (2007). Feasibility and effectiveness of cognitive‐‐behavioral therapy for posttraumatic stress disorder in preschool children: Two case reports. Journal of Traumatic Stress: Official Publication of The International Society for Traumatic Stress Studies, 20, 631–636. 10.1002/jts.20232 PMC7023908 17721975

[jcpp70121-bib-0067] Scheeringa, M.S. , Weems, C.F. , Cohen, J.A. , Amaya‐Jackson, L. , & Guthrie, D. (2011). Trauma‐focused cognitive‐behavioral therapy for posttraumatic stress disorder in three‐through six year‐old children: A randomized clinical trial. Journal of Child Psychology and Psychiatry, 52, 853–860. 21155776 10.1111/j.1469-7610.2010.02354.x PMC3116969

[jcpp70121-bib-0068] Scheeringa, M.S. , & Zeanah, C.H. (2001). A relational perspective on PTSD in early childhood. Journal of Traumatic Stress, 14, 799–815. 11776426 10.1023/A:1013002507972

[jcpp70121-bib-0069] Scheeringa, M.S. , Zeanah, C.H. , Myers, L. , & Putnam, F.W. (2003). New findings on alternative criteria for PTSD in preschool children. Journal of the American Academy of Child & Adolescent Psychiatry, 42, 561–570. 12707560 10.1097/01.CHI.0000046822.95464.14

[jcpp70121-bib-0070] Schore, A.N. (2001). The effects of early relational trauma on right brain development, affect regulation, and infant mental health. Infant Mental Health Journal, 22, 201–269.

[jcpp70121-bib-0071] Steinberg, A. , Pynoos, R. , Lieberman, A. , Osofsky, A. , & Vivrette, R. (2017). UCLA PTSD reaction index for DSM‐5 children age 6 and younger. Los Angeles, CA: University of California.

[jcpp70121-bib-0072] Struwig, E. , & Van Breda, A.D. (2012). An exploratory study on the use of eye movement integration therapy in overcoming childhood trauma. Families in Society, 93, 29–37.

[jcpp70121-bib-0073] Sveen, J. , Andersson, G. , Buhrman, B. , Sjöberg, F. , & Willebrand, M. (2017). Internet‐based information and support program for parents of children with burns: A randomized controlled trial. Burns, 43, 583–591. 28040368 10.1016/j.burns.2016.08.039

[jcpp70121-bib-0074] U.S. Department of Health and Human Services, Administration for Children and Families, Administration on Children, Youth and Families, Children's Bureau . (2025). Child maltreatment 2023.

[jcpp70121-bib-0075] Van Der Spuy, C. , & van Breda, A.D. (2019). An exploratory study on the use of eye movement integration therapy for treating trauma in early childhood in South Africa. Child Care in Practice, 25, 157–174.

[jcpp70121-bib-0077] Wherry, J.N. , Graves, L.E. , & Rhodes King, H.M. (2008). The convergent validity of the trauma symptom checklist for young children for a sample of sexually abused outpatients. Journal of Child Sexual Abuse, 17, 38–50. 19842317 10.1080/10538710701884441

[jcpp70121-bib-0078] Willheim, E. , & Schechter, D.S. (2025). Therapeutic interventions for trauma‐exposed infants, young children, and their caregivers. Child and Adolescent Psychiatric Clinics, 34, 291–310. 10.1016/j.chc.2024.08.005 40044268

[jcpp70121-bib-0079] World Health Organization . (2020). Global status report on preventing violence against children. Geneva: Author.

[jcpp70121-bib-0080] Zero to Three . (2016). DC:0–5™: Diagnostic classification of mental health and developmental disorders of infancy and early childhood. Washington, DC: Author.

